# Effects of dietary diversity on frailty in Chinese older adults: a 3-year cohort study

**DOI:** 10.1186/s12877-023-03875-5

**Published:** 2023-03-14

**Authors:** Ying Duan, Qi Qi, Yan Cui, Ling Yang, Min Zhang, Huaqing Liu

**Affiliations:** 1 School of Public Health, Bengbu Medical College, No.2600 Donghai Ave, Bengbu, 233030 China; 2 School of Health Management, Bengbu Medical College, Bengbu, Anhui China

**Keywords:** Dietary diversity, Frailty, China, Older adults

## Abstract

**Background:**

Frailty has emerged as a global health burden with increased population aging. A diverse diet is essential for an adequate and balanced supply of nutrients. However, limited evidence supports the relationship between dietary diversity and frailty. We therefore assessed the associations of dietary diversity with the risk of frailty.

**Methods:**

We used the Chinese Longitudinal Healthy Longevity Survey to analyze a prospective cohort of Chinese older adults. A total of 1948 non-frail older adults were included in the final sample. Participants were categorized into groups with high or low dietary diversity scores (DDSs) using a food frequency questionnaire. A Generalized Estimating Equation were used to estimate risk ratios (RRs) and 95% confidence intervals (CIs) for determining frailty incidence.

**Results:**

Among 1,948 participants, 381 had frailty with the prevalence of 19.56% during the 3-year follow-up period. Compared with the low DDS group, the high DDS group exhibited a lower risk of frailty (RR, 0.72; 95% CI: 0.57–0.91). Compared with those with a consistently low DDS, the RR of participants with a consistently high DDS for frailty was 0.56 (95% CI: 0.42–0.74). Moreover, meat, beans, fish, nuts, fresh fruits, and fresh vegetables were inversely associated with frailty. In stratified analysis, a consistently high DDS, compared with a consistently low DDS, reduced the risk of frailty for people aged 65-79 years and those living in town and rural areas.

**Conclusion:**

This study found a prospective association between dietary diversity and frailty among Chinese older adults. These findings stressed that it is important to improve dietary diversity for older adults to promote healthy ageing, particularly for young older adults and in town and rural areas.

**Supplementary Information:**

The online version contains supplementary material available at 10.1186/s12877-023-03875-5.

## Introduction

The number of older adults aged more than 65 years has increased from 461 million in 2004 to an estimated 2 billion in 2050 [1]. Frailty has emerged as a global health burden, and the incidence of frailty is likely to increase under population aging [2]. The prevalence of frailty in the elderly individuals ranges from 12 to 24% [3]. Frailty is associated with a range of adverse outcomes, including morbidity, mortality, and increased health care costs [4, 5], and it has major implications for clinical practice and public health.

Frailty is defined as increased vulnerability to stressors across multiple bodily systems [6], including cognitive, psychosocial, and physical components [7]. Frailty is dynamic process that deteriorates or improves over time [8]. Strategies for preventing and delaying the progression of frailty are crucial [9].

Nutrition, a modifiable factor for frailty [6], plays a critical role in causing, mediating, and reversing frailty in older adults [10]. Various foods and nutrients have been reported to assist in preventing frailty. Cross-sectional studies in China [11], the United States [12], and the United Kingdom [13] have revealed that fruit and vegetable consumption is associated with a reduced risk of frailty. A prospective study [14] of elderly Japanese reported that higher baseline dairy and milk consumption was associated with a lower risk of frailty. Another prospective cohort study [15] in Spain also revealed that an increased intake of yogurt milk and low-fat was associated with a lower incidence of frailty. A meta-analysis of 10 studies reported a lower frailty prevalence among older adults with high protein intake than among older adults with low protein intake [16]. A systematic review of longitudinal data on vitamin D and frailty indicated an association between lower vitamin D intake and a higher risk of frailty [17].

Nutrition is a key factor for the prevention and treatment of frailty. However, a single nutrient or food cannot reflect the nutritional status of an individual in real life. Few studies have explored the effects of individual nutrition on frailty from a holistic perspective. Dietary diversity is defined as the number of different food groups or foods consumed in a given period, and it ensures a rich provision of macronutrients and micronutrients [18]. A diverse diet is essential for an adequate and balanced supply of nutrients. Dietary diversity score (DDS), as an indicator of dietary diversity, can be applied to all age groups [19]. Two cross-sectional studies [20, 21] in Japan have reported that DDS may be associated with frailty in older adults. The present 3-year cohort study explored the association between DDS and frailty among Chinese older adults.

## Materials

### Study population

The Chinese Longitudinal Healthy Longevity Survey (CLHLS) is a nationwide, prospective cohort study of community-dwelling older adults in China. The survey has been conducted in 23 counties and cities randomly selected from 31 provinces, and the population of these areas covers 85% of China's population. The survey was carried out in 1998, and follow-up surveys are conducted every 2 to 3 years. The trained staff interviewed the elders face to face and systematically collected their information. [22].

**Fig. 1:**
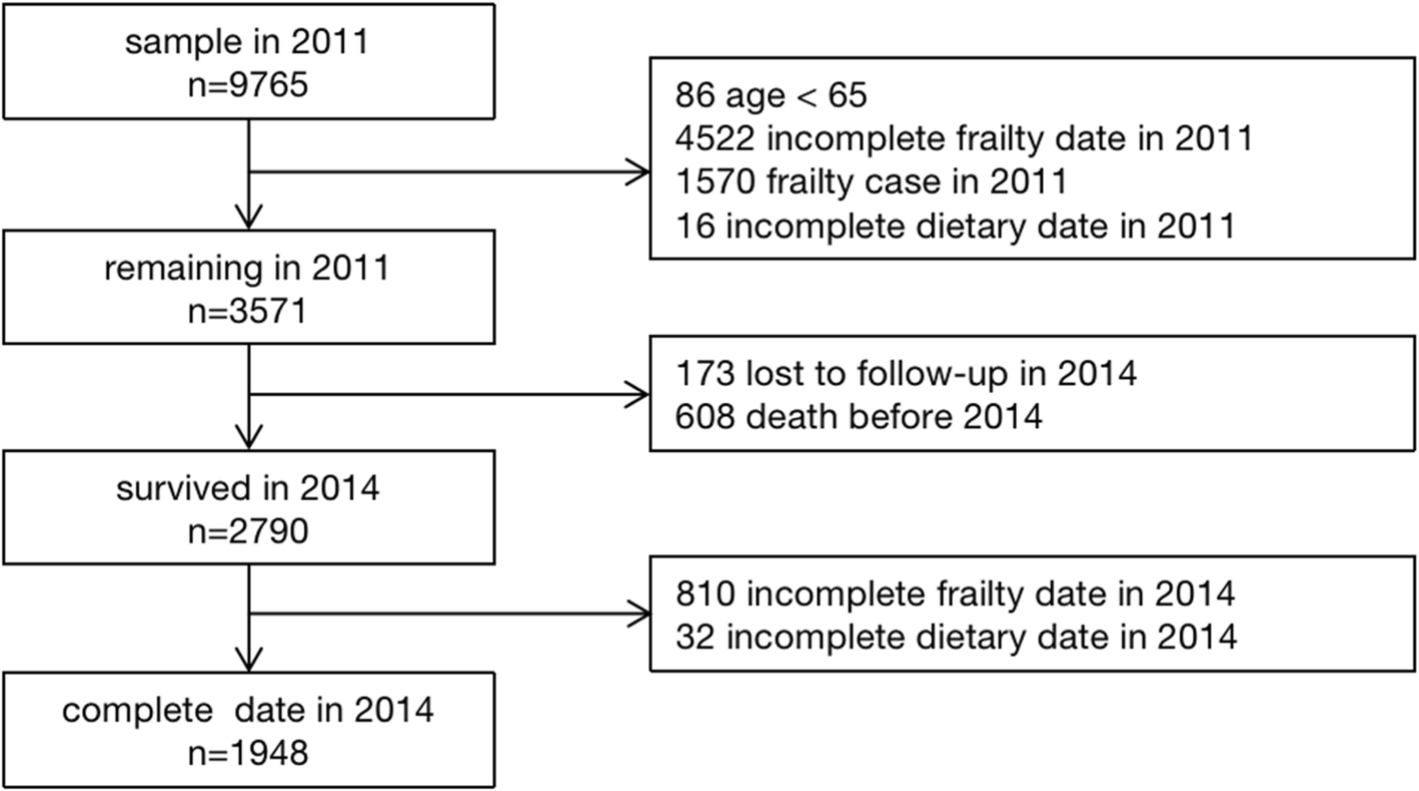
Flowchart of the participant selection process

Our study used data for the period from 2011 to 2014. 9765 individuals participated in baseline interviews between 2011 and 2012. We excluded participants aged less than 65 years (*n* = 86) in 2011. Furthermore, from this sample, we excluded participants who presented with frailty in 2011 (*n* = 1570), those with missing information relating to frailty (*n* = 4522) and those with missing values related to DDS (*n* = 16). We also excluded participants who were lost or dead. Finally, we analyzed the data of 1948 individuals from 2011 to 2014 to determine the relationship between DDS and frailty. Figure 1 depicts the flowchart of the patient selection process in this study. The missing participants were more likely to be female, aged 80 years or above, financially dependent, of informal education, of other marital status, of underweight, and to live in town and rural areas and to not smoke, not drink, not exercise (Table S1).

### Assessment of frailty

The frailty index (FI) counts deficits in health to assessment of frailty [23]. According to a study [24] measuring frailty, the FI contains items related to 44 health deficits, including activities of daily living (basic and instrumental), chronic diseases, and psychological function. The FI is calculated by adding all deficits and dividing the sum by the total number of deficits. Detailed information on the calculation procedure is presented in the Table S2. In the present study, the FI reflected cumulative health deficits, and it is comparable to the indices used in other CLHLS-based studies [25–27] and those used in studies conducted in the United States [28], Canada [29], and Hong Kong [30]. The FI is a continuous variable and ranges from 0 to 1; a higher value indicates a higher degree of frailty. According to the FI, participants were divided into two groups: non-frailty group (FI ≤ 0.21) and frailty group (FI > 0.21) [31].

### Assessment of dietary diversity

The participants completed a food frequency questionnaire [32] on 11 major food groups or items: meat, fish, eggs, beans, mushrooms or algae, tea, garlic, milk products, nuts, fresh vegetables, and fresh fruits; the questionnaire was used to determine their DDS during a face-to-face interview. Because almost all Chinese people consume cereals and oil daily, we did not include these two food groups in the DDS questionnaire [33]. The DDS questionnaire we used was primarily composed of items on healthy food. It was created to assess the adequacy of food consumption and the healthiness of diets, and its scientific validity has been demonstrated [34].

DDS was calculated according to the frequency of intake of the 11 food groups. The scoring criteria and intake frequencies are detailed in the Table S3. The total DDS was calculated as the sum of the scores of the 11 food groups, with the highest and lowest scores being 11 and 0, respectively. The higher was the score, the greater was the dietary diversity. We divided the participants into two groups according to the median DDS, namely the low and high DDS groups.

We also classified changes in dietary diversity (CDD) from 2011 to 2014 into the following four categories: declining dietary diversity, improving dietary diversity, consistently low dietary diversity, and consistently high dietary diversity (Table S4).

### Assessment of covariates

According to previous research [35], frailty is influenced by older people’s individual heterogeneity, socioeconomic level, and health status. Therefore, to account for crucial differences, we evaluated various baseline characteristics. Covariates included age group (65–79 or ≥ 80 years), sex (male or female), body mass index (BMI; underweight, normal, overweight, or obese), residential location (urban or town and rural), and marital status (currently married and living with spouse or other). Education status was classified into formal education [≥ 1 year(s) of education] and informal education (< 1 year of education). Drinking, smoking, and exercise status was defined according two questions (“drink/smoke/exercise or not at present?” and “drink/smoke/exercise or not in the past?”). It was defined as no if participants answer no to both questions; otherwise, was defined as yes. The participants were regarded as having a history of chronic disease if they self-reported hypertension, heart disease, bronchitis, asthma, emphysema, pneumonia, or diabetes. We distinguished financial support into financial independence and dependence. We regarded financial independence as receiving a work or retirement wage, and financial dependence as financially relying on other family members.

### Statistical analysis

Independent chi-square tests were used to examine the initial basic characteristics of the groups (sex, age, residential location, education status, BMI, drinking status, smoking status, exercise status, marital status, financial support, and history of chronic disease). A Generalized Estimating Equation (GEE) were used to determine the relationship between dietary diversity and frailty incidence. In addition, we conducted a stratified analysis based on age and residential location.

For all outcomes, we constructed models without any adjusted covariates (Model 1), models adjusted only for sex and age (Model 2), and models further adjusted for residential location, education status, BMI, drinking status, smoking status, exercise status, marital status, financial support, and history of chronic disease (Model 3). The sampling weight variables in CLHLS are calculated based on the age-sex-residence-specific distribution of the population. Our analysis results were weighted to ensure its representativeness.

Given that large samples losses occur due to incomplete frailty data, we conducted a sensitivity analysis. If the missing value in the 44 health deficits of participants is less than or equal to 5, these participants will not be excluded. Given pre-frailty older adults may be change dietary behaviors, we conducted another sensitivity analysis and examined the relationship between frailty and dietary diversity after excluding subjects with pre-frailty at baseline.

Statistical analysis was conducted using R studio, version 4.1.2 (R Foundation for Statistical Computing). Statistical significance was defined as a two-sided *P* value threshold of 0.05.

## Results

### Baseline characteristics

Table 1 provides the baseline characteristics of the study participants. The sample was composed of 1948 participants, with 1040 men (53.4%) and 908 women (46.6%). Among all participants, 43.0% were 80 years and older, 79.6% lived in town and rural areas, 48.2% were currently married and living with their spouse, 44.3% had informal education, 38.9% were financially independent, and 57.3% had no chronic illnesses.

**Table 1:** Baseline characteristics of participants without frailty

Characteristics	*N (%)*	Low DDS (weighted *%*)	High DDS (weighted *%*)	*X*^*2*^
**Total**	1948	45.7	54.3	
**Age(years)**				21.76*
65–79	1111(57.0)	44.3	55.7	
≥ 80	837(43.0)	55.7	44.3	
**Sex**				32.78*
Male	1040(53.4)	41.3	58.7	
Female	908(46.6)	50.5	49.5	
**Residential location**				61.64*
Town and rural	1550(79.6)	48.3	51.7	
Urban	398(20.4)	30.3	69.7	
**Education**				132.62*
Informal education	863(44.3)	58.4	41.6	
Formal education	1083(55.7)	38.9	61.1	
**Financial support**				217.51*
Financial dependence	1190(61.1)	55.9	44.1	
Financial independence	757(38.9)	31.9	68.1	
**Marital status**				23.67*
Currently married and living with spouse	937(48.2)	55.6	44.4	
Other	1008(51.8)	40.6	59.4	
**Smoking status**				0.36
No	1106(56.9)	44.7	55.3	
Yes	837(43.1)	45.7	54.3	
**Drinking status**				10.39*
No	1185(61.2)	47.9	52.1	
Yes	750(38.8)	42.7	57.3	
**Exercise status**				71.20*
No	720(37.0)	53.7	46.3	
Yes	1222(63.0)	39.5	60.5	
**Body mass index (kg/m2)**				197.00*
Underweight (< 18.5)	330(17.1)	70.8	29.2	
Normal (18.5–23.99)	1075(55.6)	45.8	54.2	
Overweight (24–27.99)	399(20.6)	32.0	68.0	
Obese (≥ 28)	131(6.8)	43.2	56.8	
**Chronic disease**				7.60*
No	1116(57.3)	43.9	56.1	
Yes	832(42.7)	48.4	51.6	

*DDS* Dietary diversity score

^*^P < 0.05

The mean score of the DDS was 5.7 with the standard deviation of 1.8. From 2011 to 2014, 608 (21.2%) participants maintained low DDS, 279 (14.3%) participants changed from high DDS to low DDS, 328 (16.8%) participants changed from low DDS to high DDS, and 733 (37.6%) participants maintained high DDS.

The participants who are aged 65–79, female, live in urban areas, were overweight, have formal education, have another marital status, be financially independent, be physically active drink, do not smoke and have no history of chronic diseases were more likely to have high DDS.

### Association of DDS with frailty prevalence

Among 1,948 participants, 381 had frailty with the prevalence of 19.56% during the 3-year follow-up period. These cases involved 208 participants with a low DDS and 173 participants with a high DDS. Overall, the crude rate of frailty events was higher in the low DDS group than in the high DDS group (Table 2). In the unadjusted analysis, the risk ratio (RR) of the participants with a high DDS for frailty was 0.61 [95% confidence interval (CI): 0.50–0.76] compared with that of the participants with a low DDS. Following adjustment for age, sex, residential location, education status, BMI, drinking status, smoking status, exercise status, marital status, financial support, and history of chronic disease (Model 3), the inverse association was still significant (RR, 0.72; 95% CI: 0.57–0.91).

**Table 2:** Association between DDS and frailty

**Characteristics**	Model 1 RR (95% CI)	Model 2 RR (95% CI)	Model3 RR (95% CI)
**DDS as continuous variable**			
	0.83(0.78,0.88)*	0.86(0.81,0.92)*	0.88(0.82,0.94)*
**DDS as categorical variable (ref. = Low DDS)**			
High DDS	0.61(0.50,0.76)*	0.69(0.55,0.85)*	0.72(0.57,0.91)*

*Model 1: no adjustment; Model 2: adjusted for age and sex; Model 3: adjusted for age, sex, residential location, education status, body mass index, drinking status, smoking status, exercise status, marital status, financial support, and history of chronic disease*

*DDS* Dietary diversity score, *CI* Confidence interval, *RR* Risk ratio

^*^P < 0.05

When DDS was adopted as a continuous variable, this association did not change (RR, 0. 83; 95% CI: 0.78–0.88 in Model 1; RR, 0.88; 95% CI: 0.82–0.94 in Model 3).

### Association of CDD with frailty prevalence

The association between CDD and frailty is presented in Table 3. Compared with those with a consistently low DDS, the RR of the participants with a consistently high DDS for frailty was 0.46 (95% CI: 0.35–0.59) in the crude model. Following adjustment for all the covariates, the inverse association between a consistently high DDS and frailty remained significant (*P* < 0.05).

**Table 3:** Association between CDD and frailty

	RR (95%CI)			
	Consistently Low Dietary Diversity	Declining Dietary Diversity	Improving Dietary Diversity	Consistently High Dietary Diversity
Model 1	1 (reference)	0.80(0.58,1.09)	0.68(0.49,0.92)*	0.46(0.35,0.59)*
Model 2	1 (reference)	0.86(0.62,1.18)	0.73(0.53,1.01)	0.53(0.41,0.70)*
Model 3	1 (reference)	0.94(0.67,1.33)	0.79(0.56,1.11)	0.56(0.42,0.74)*

*Model 1: no adjustment; Model 2: adjusted for age and sex; Model 3: adjusted for age, sex, residential location, education status, body mass index, drinking status, smoking status, exercise status, marital status, financial support, and history of chronic disease*

*CDD* Changes in dietary diversity, *CI* Confidence interval, *RR* Risk ratio

^*^P < 0.05

In stratified analysis (Table 4), a consistently high DDS, compared with a consistently low DDS, reduced the risk of frailty for people aged 65–79 years (RR, 0.46; 95% CI: 0.33–0.64) and those living in town and rural areas (RR, 0.46; 95% CI: 0.33–0.64) after adjusting for all covariates, but not for people aged 80 years and older (RR, 1.00; 95% CI: 0.56–1.80) and those living in urban areas (RR, 1.38; 95% CI: 0.48–3.93).

**Table 4:** Associations between CDD and frailty, stratified by age and residential location

Characteristics	Final model RR (95% CI)			
	Consistently Low Dietary Diversity	Declining Dietary Diversity	Improving Dietary Diversity	Consistently High Dietary Diversity
**Stratified by age**^**a**^				
65–79	Reference	0.84(0.57,1.25)	0.80(0.55,1.17)	0.46(0.33,0.64)*
≥ 80	Reference	1.50(0.73,3.06)	0.68(0.33,1.44)	1.00(0.56,1.80)
**Stratified by residential location**^**b**^				
Town and rural	Reference	0.94(0.65,1.35)	0.73(0.50,1.05)	0.46(0.33,0.64)*
Urban	Reference	1.62(0.45,5.92)	2.34(0.71,7.70)	1.38(0.48,3.93)

*CDD* Changes in dietary diversity, *CI* Confidence interval, *RR* Risk ratio

^a^Adjusted for sex, residential location, education status, body mass index, drinking status, smoking status, exercise status, marital status, financial support, and history of chronic disease

^b^Adjusted for age, sex, education status, body mass index, drinking status, smoking status, exercise status, marital status, financial support, and history of chronic disease

^*^P < 0.05

In the two sensitivity analyses, this association between dietary diversity and frailty remains unchanged (Table S5 and Table S6).

### Association of single specific foods with frailty prevalence

**Fig. 2:**
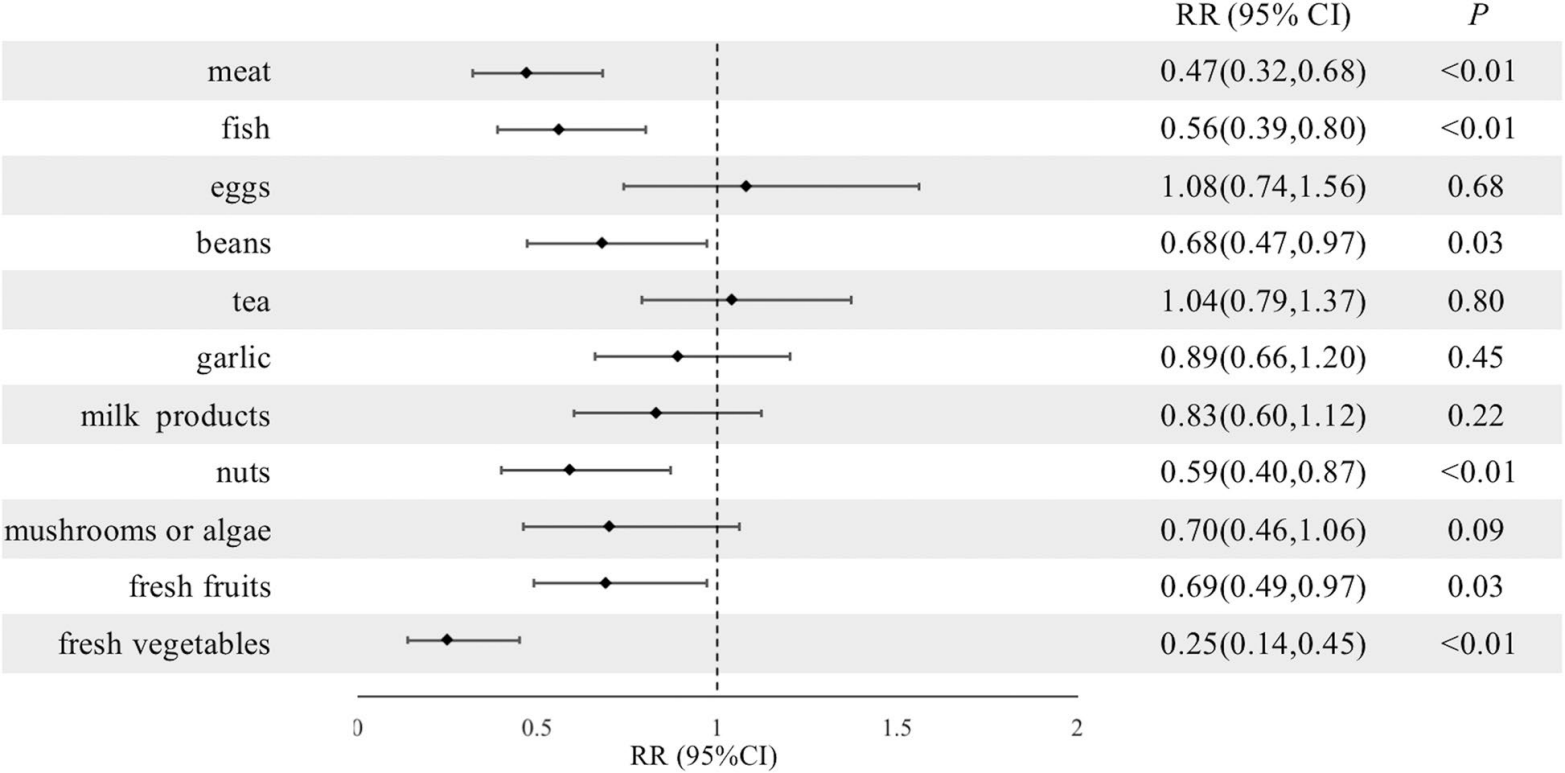
RRs and 95% CIs indicating the associations between single food groups and frailty. CI: confidence interval, RR: risk ratio. Adjusted for age, sex, residential location, education status, body mass index, drinking status, smoking status, exercise status, marital status, financial support, and history of chronic disease

We adopted the occurrence of frailty as the dependent variable and the intake of the 11 major food groups in 2011 as the independent variable in GEE. Meat (RR, 0.47; 95% CI: 0.32–0.68), fish (RR, 0.56; 95% CI: 0.39–0.80), beans (RR, 0.68; 95% CI: 0.47–0.97), nuts (RR, 0.59; 95% CI: 0.40–0.87), fresh fruits (RR, 0.69; 95% CI: 0.49–0.97) and fresh vegetables (RR, 0.25; 95% CI: 0.14–0.45) was inversely associated with frailty following adjustment for all covariates. Most of the associations between other single food groups and frailty were in the expected direction (Fig. 2).

## Discussion

From a holistic nutritional perspective, this study evaluated a prospective association of dietary diversity with frailty in a Chinese older adult population. Our study revealed that a high DDS reduced the incidence of frailty. In addition, the participants with a consistently high DDS had a lower risk of frailty than those with a consistently low DDS during the 3-year follow-up period. Whether the participants with pre-frailty at baseline were excluded or the participants with some missing values (≤ 5) of the health deficit were remained, dietary diversity showed a protective effect on frailty of older adults, which to some extent showed the stability of our results. This study further verified the relationship between dietary diversity and frailty and provided new evidence that can be applied for preventing or delaying frailty development in older adults.

A high DDS is associated with an adequate intake of nutrients and a favorable nutritional status. A low DDS is related to the risk of undernutrition [36], which is characterized by inadequate nutrient intake and reduced energy reserves. Obesity may be one of the risk factors for frailty [37]. However, this association remains contradictory, and another research showed that obesity is related to reducing the risk of frailty in multivariate analysis [38]. Interestingly, in our study, overweight subjects are more likely to have high DDS. These findings indicated that the relationship between obesity, frailty and DDS should be further investigated in future study. Energy and nutrient deficiencies may affect mitochondrial function and induce muscle-related symptoms, including frailty [39]. Malnourished older adults have a high prevalence of frailty [40]. Additionally, the participants with a high DDS had a high intake of protein, vitamins, and antioxidant nutrients [41, 42], all of which have been reported to the prevention of frailty. Loss of muscle mass and strength is regarded as a key pathology leading to frailty [43]. Adequate protein intake helps to maintain muscle function in older adults [44]. A high DDS can reduce inflammation and oxidative stress [45], both of which accelerate the loss of muscle and bone mass and the deterioration of central nervous system function [46–48], leading to frailty. The effects of many nutrients depend on the presence of other nutrients in different food groups, and only through a high dietary diversity can nutritionally balance and disease prevention be achieved [49]. For example, food protein sources are crucial, and vitamins and minerals in fruits and vegetables are also essential for the synthesis of muscle protein [20]. If a diet lacks diversity, nutrients that contribute to frailty prevention are ineffective. Nutrient interactions (i.e., their balance) are more critical in health and aging than nutrients acting alone [50]. In addition, eating a variety of foods throughout the day requires health awareness in the performance of activities such as shopping, cooking, and meal planning. These intentional instrumental activities may effectively assist individuals in maintaining functional ability [51] and physical performance [52]. Moreover, studies have indicated that a diverse diet can promote a healthier gut microbiome [53], which plays a role in the anabolic resistance of skeletal muscle to dietary proteins [54] and may play a role in the prevention of frailty.

To understand the relationship between dietary diversity and frailty, the effects of long-term dietary behaviors on frailty were explored in this study. Our study revealed that a consistently high DDS can reduce the risk of frailty among Chinese older adults following adjustment for confounding factors. Older adults may experience a decline in their chewing ability as a result of aging [55], and chewing ability is associated with dietary diversity [56]. Therefore, the dietary diversity of older adults may change in the future. Perhaps only the long-term rather than the short-term maintenance of high dietary diversity has a beneficial effect on frailty. Consistent with our findings, our previous study [57] demonstrated the protective effect of long-term tea consumption on frailty in older adults.

The town and rural population differ from urban dwellers in eating habits and conditions [58]. In urban areas, residents with low dietary diversity may indirectly indicate a reduced ability to go out, leading to fewer opportunities to replenish the pantry; however, in town and rural areas, dietary diversity may reflect different dietary choices. Urban areas have more complete food supply systems and better infrastructure, and urban residents have higher food diversity and availability than rural residents [59, 60]. In our study, consistently high dietary diversity was associated with a lower risk of frailty for town and rural residents, but not for older adults living in urban areas. With the increase in age, the prevalence of frailty increases [61] and the ability to chew and digest decreases. Decreased chewing and digestion due to aging will lead to lower DDS [62]. This may explain the protective effect of consistently high DDS against frailty in older adults aged 65–79, but not in those aged 80 or older.

We further explored the associations between specific food groups and frailty, and the results revealed the positive effect of meat, beans, fish, nuts, fresh fruits, and fresh vegetables on the prevention of frailty. Fruits and vegetables are rich sources of antioxidants such as carotene and vitamin C [63]. Meat and fish are rich in protein, which increases muscle synthesis [64]. Nuts and legumes are good sources of vegetable protein, which prevents muscle mass loss [65]. These are all related to preventing frailty [43, 48, 66]. Milk products are rich in calcium, which may be related to the prevention of osteoporosis and then to reduce frailty. Some research results also show that calcium intake is related to frailty [37, 67]. In our study, milk products have slight trend on the reduction of the risk of frailty, although this reduced effect did not reach statistical significance.

These findings stressed that public health worker should take actions or interventions on diet to reduce the incidence of frailty in older adults. Dietary diversity should be recommended. It is important to strengthen healthy dietary behaviors education for older adults and caregivers to increase the awareness of dietary diversity. Moreover, the community can strengthen diverse foods supplies for older adults to meet their needs of dietary diversity, particularly for young older adults and in town and rural areas.

To the best of our knowledge, this study is the first to examine the association between DDS and frailty in Chinese older adults using nationally representative cohort data. An advantage of the study is its exploration of the role of ongoing dietary diversity. Our study has some limitations. First, self-reported information collected using the food frequency questionnaire is prone to recall bias. In addition, this questionnaire only collected information on the frequency of food intake and not the specific amount of food intake. Finally, the included participants were more likely to be male, aged 65—79 years, currently married and living with spouse, financially dependent, of formal education, of normal BMI, and to live in town and rural areas and to not smoke, not drink, exercise. Differences in baseline characteristics between the lost and included samples showed that our study may have selection bias. And these may affect the robustness of our results to some extent. For example, the prevalence of frailty increases with the increase of age [61], and dietary patterns may also change with aging. Studies have also shown that frailty occurs more often in women than in men [68].

## Conclusion

This study found a prospective association between dietary diversity and frailty among Chinese older adults.

## Supplementary Information

**Additional file 1: Table S1. **Baseline characteristics of lost sample and final sample. **Table S2.** Scoring criteria for frailty. **Table S3.** Scoringcriteria for dietary diversity. **Table S4. **Changes in dietary diversity. **Table S5. **Association between dietary diversity and frailty after retaining participants with some health defect absence value (≤5). **Table S6. **Association between dietary diversity and frailty after excluding participants with pre-frailty in 2011. 

## Data Availability

Data are from the Chinese Longitudinal Healthy Longevity Survey, which is a public, open access repository (https://opendata.pku.edu.cn/dataverse/CHADS).

## Abbreviations

CLHLS: Chinese Longitudinal Healthy Longevity Survey
DDS: Dietary diversity scores
GEE: Generalized Estimating Equation
RR: Risk ratio
CI: Confidence interval
FI: Frailty index
CDD: Changes in dietary diversity
BMI: Body mass index
