# Supplementary material for: Effects of dietary diversity on frailty in Chinese older adults: a 3-year cohort study
Source: BMC Geriatr. 2023 Mar 14;23:141. doi: 10.1186/s12877-023-03875-5 (PMC10012609; doi:10.1186/s12877-023-03875-5)
Supplement: Supplementary file 1 — Additional file 1: Table S1. Baseline characteristics of lost sample and final sample. Table S2. Scoring criteria for frailty. Table S3. Scoringcriteria for dietary diversity. Table S4. Changes in dietary diversity. Table S5. Association between dietary diversity and frailty after retaining participants with some health defect absence value (≤5). Table S6. Association between dietary diversity and frailty after excluding participants with pre-frailty in 2011. [file 12877_2023_3875_MOESM1_ESM.docx]

**Table S1:** Baseline characteristics of lost sample and final sample.

| **Characteristics** | ***N (%)*** | **Lost sample** | **Final sample** | Statistics |
| --- | --- | --- | --- | --- |
| **Total** | 9765 | 7817 | 1948 |  |
| **DDS,** M (SD) |  | 5.3(1.8) | 5.7(1.8) | -9.20* |
| **Age(years)** |  |  |  | 627.67* |
| 65-79 | 3235(33.1) | 2124(27.2) | 1111(67.0) |  |
| ≥80 | 6530(66.9) | 5693(72.8) | 837(43.0) |  |
| **Sex** |  |  |  | 68.54* |
| Male | 4398(45.0) | 3358(43.0) | 1040(53.4) |  |
| Female | 5367(55.0) | 4459(57.0) | 908(46.6) |  |
| **Residential location** |  |  |  | 11.82* |
| Town and rural | 8030(82.2) | 6480(82.9) | 1550(79.6) |  |
| Urban | 1735(17.8) | 1337(17.1) | 398(20.4) |  |
| **Education** |  |  |  | 189.71* |
| Informal education | 5652(58.1) | 4789(61.6) | 863(44.3) |  |
| Formal education | 4072(41.9) | 2989(38.4) | 1083(55.7) |  |
| **Financial support** |  |  |  | 149.95* |
| Financial dependence | 6991(72.2) | 5801(75.0) | 1190(61.1) |  |
| Financial independence | 2688(27.8) | 1931(25.0) | 757(38.9) |  |
| **Marital status** |  |  |  | 245.83* |
| Currently married and living with spouse | 3532(36.5) | 2524(32.7) | 1008(51.8) |  |
| Other | 6138(63.5) | 5201(67.3) | 937(48.2) |  |
| **Smoking status** |  |  |  | 86.75* |
| No | 6369(65.9) | 5263(68.1) | 1106(56.9) |  |
| Yes | 3299(34.1) | 2462(31.9) | 837(43.1) |  |
| **Drinking status** |  |  |  | 55.71* |
| No | 6575(68.3) | 5390(70.1) | 1185(61.2) |  |
| Yes | 3052(31.7) | 2302(29.9.) | 750(38.8) |  |
| **Exercise status** |  |  |  | 295.67* |
| No | 5226(54.4) | 4506(58.8) | 720(37.1) |  |
| Yes | 4375(45.6) | 3153(41.2) | 1222(62.9) |  |
| **Body mass index (kg/m2)** |  |  |  | 99.14* |
| Underweight (<18.5) | 2241(24.9) | 1911(27.1) | 330(17.1) |  |
| Normal (18.5–23.99) | 4780(53.1) | 3705(52.5) | 1075(55.6) |  |
| Overweight (24–27.99) | 1441(16.0) | 1042(14.8) | 399(20.6) |  |
| Obese (≥28) | 536(6.0) | 405(5.7) | 131(6.8) |  |
| **Chronic disease** |  |  |  | 0.99 |
| No | 5223(56.3) | 4107(56.0) | 1116(57.3) |  |
| Yes | 4055(43.7) | 3223(44.0) | 832(42.7) |  |

**P* < 0.05; DDS: dietary diversity score**.**

**Table S2:** Scoring criteria for frailty.

| Variables | Values |
| --- | --- |
| Feel useless with age | Never =0; seldom =0.25; sometimes =0.5; often =0.75; always=1 |
| Feel lonely and isolated | Never =0; seldom =0.25; sometimes =0.5; often =0.75; always=1 |
| Feel fearful or anxious | Never =0; seldom =0.25; sometimes =0.5; often =0.75; always=1 |
| Keep my belongings neat and clean | Always =0; often =0.25; sometimes =0.5; seldom =0.75; never=1 |
| Self-reported health | Very good =0; good =0.25; so so=0.5; bad =0.75; very bad =1 |
| Do you feel any change in your health since the last year? | Much better =0; slightly better =0.25; almost the same =0.5; slightly worse =0.75; much worse=l |
| Make own decision | Always =0; often =0.25; sometimes =0.5; seldom =0.75; never=1 |
| Bathing | Without assistance =0; partial assistance =0.5; need assistance=1 |
| Dressing | Without assistance =0; partial assistance =0.5; need assistance =1 |
| Toileting | Without assistance =0; partial assistance =0.5; need assistance=1 |
| Transferring | Without assistance =0; partial assistance =0.5; need assistance = 1 |
| Continence | Without assistance =0; partial assistance =0.5; need assistance =1 |
| Feeding | Without assistance =0; partial assistance =0.5; need assistance =1 |
| Able to go outside to visit neighbors? | Yes =0; a little difficult =0.5; not able to do so=l |
| Able to go shopping by yourself? | Yes =0; a little difficult =0.5; not able to do so=l |
| Able to make food by yourself? | Yes =0; a little difficult =0.5; not able to do so=1 |
| Able to wash clothes by yourself? | Yes =0; a little difficult =0.5; not able to do so=1 |
| Able to walk I km? | Yes =0; a little difficult =0.5; not able to do so=l |
| Able to carry 5-kg weight? | Yes =0; a little difficult =0.5; not able to do so=l |
| Able to crouch and stand for three times? | Yes =0; a little difficult =0.5; not able to do so =1 |
| Able to take public transport? | Yes =0; a little difficult =0.5; not able to do so=1 |
| Visual function | Can see and distinguish the break in the circle =0; can see but not distinguish the break in the circle =0.33; cannot see =0.67; blind =1 |
| Hand behind neck | Both =0; right =0.5; left =0.5; neither=1 |
| Hand behind lower back | Both =0; right =0.5; left =0.5; neither=1 |
| Raise arms upright | Both =0; right =0.5; left =0.5; neither=1 |
| Able to stand up from sitting in a chair | Yes, without using hands =0; yes, using hands =0.5; no =1 |
| Able to pick up a book from the floor | Yes, without using hands =0; yes, using hands =0.5; no =1 |
| Of times suffering from serious illness in the past 2 years | Not applicable =0; one serious illness =l; two or more serious illnesses =2 |
| Suffering from hypertension? | No =0; yes=1 |
| Suffering from diabetes? | No =0; yes=1 |
| Suffering from heart disease? | No =0; yes=1 |
| Suffering from stroke or cardiovascular disease? | No =0; yes=l |
| Suffering from bronchitis, emphysema, pneumonia, and asthma? | No =0; yes=l |
| Suffering from tuberculosis? | No =0; yes=l |
| Suffering from cataract? | No =0; yes=l |
| Suffering from cancer? | No =0; yes=1 |
| Suffering from glaucoma? | No =0; yes=l |
| Suffering from gastric or duodenal ulcer? | No =0; yes=1 |
| Suffering from Parkinson's disease? | No =0; yes=1 |
| Suffering from bedsore? | No =0; yes=l |
| Suffering from arthritis? | No =0; yes=1 |
| Suffering from dementia? | No =0; yes=l |
| Was interviewee able to hear? | Yes, without hearing aid =0; yes, but needs hearing aid =0.33; partly, despite using hearing aid =0.67; no=1 |
| The health of interviewee rated by interviewer | Surprisingly healthy =0; relatively healthy =0.33; moderately ill =0.67; very ill=l |

**Table S3:** Scoring criteria for dietary diversity.

| Food Group | DDS |
| --- | --- |
| Fresh fruit | “Almost every day” = 1 |
|  | “Quite often” = 0.75 |
| Fresh vegetable | “Occasionally” = 0.5 |
|  | “Rarely or never” = 0 |
| Meat |  |
| Fish |  |
| Eggs | “Almost every day” = 1 |
| Bean products | “At least once a week” = 0.75 |
| Garlic | “At least once a month” = 0.5 |
| Milk products | “Occasionally” = 0.25 |
| Nut products | “Rarely or never” = 0 |
| Mushrooms or algae |  |
| Tea |  |

**Table S4:** Changes in dietary diversity.

| CDD | DDS in 2011 | DDS in 2014 |
| --- | --- | --- |
| Consistently low dietary diversity (low-low) | <5.7 | <5.7 |
| Declining dietary diversity (high-low) | ≥5.7 | <5.7 |
| Improving dietary diversity (low-high) | <5.7 | ≥5.7 |
| Consistently high dietary diversity (high-high) | ≥5.7 | ≥5.7 |

**Table S5:** Association between dietary diversity and frailty after retaining participants with some health defect absence value (≤5).

| **Characteristics** | Model 1  RR (95% CI) | Model 2  RR (95% CI) | Model3  RR (95% CI) |
| --- | --- | --- | --- |
| **DDS as continuous variable** | | | |
|  | 0.84 (0.81,0.86)* | 0.88(0.83,0.92)* | 0.88(0.83,0.93)* |
| **DDS as categorical variable (ref.=Low DDS)** | | | |
| High DDS | 0.57 (0.48,0.68)* | 0.64 (0.54,0.77)* | 0.64 (0.53,0.78)* |
| **CDD (ref.= Consistently Low Dietary Diversity)** | | | |
| Declining Dietary Diversity | 0.84(0.66,1.08) | 0.91(0.70,1.17) | 0.99(0.75,1.28) |
| Improving Dietary Diversity | 0.64(0.49,0.83)* | 0.67(0.51,0.88)* | 0.69(0.52,0.92)* |
| Consistently High Dietary Diversity | 0.38(0.31,0.48)* | 0.45(0.36,0.56)* | 0.42(0.33,0.53)* |

**P* < 0.05; DDS: dietary diversity score; CI: confidence interval; RR: risk ratio; CDD: changes in dietary diversity.

Model 1: no adjustment; Model 2: adjusted for age and sex; Model 3: adjusted for age, sex, residential location, education status, body mass index, drinking status, smoking status, exercise status, marital status, financial support, and history of chronic disease.

**Table S6:** Association between dietary diversity and frailty after excluding participants with pre-frailty in 2011.

| **Characteristics** | Model 1  RR (95% CI) | Model 2  RR (95% CI) | Model3  RR (95% CI) |
| --- | --- | --- | --- |
| **DDS as continuous variable** | | | |
|  | 0.83(0.73,0.94)* | 0.86(0.76,0.97)* | 0.84(0.73,0.97)* |
| **DDS as categorical variable (ref.=Low DDS)** | | | |
| High DDS | 0.72(0.50,1.16) | 0.80(0.54,1.19) | 0.77(0.51,1,16) |
| **CDD (ref.= Consistently Low Dietary Diversity)** | | | |
| Declining Dietary Diversity | 0.78(0.45,1.32) | 0.91(0.70,1.17) | 0.85(0.49,1.46) |
| Improving Dietary Diversity | 0.31(0.15,0.54)* | 0.67(0.51,0.88)* | 0.32(0.15,0.67)* |
| Consistently High Dietary Diversity | 0.43(0.27,0.68)* | 0.49(0.31,0.77)* | 0.48(0.30,0.77)* |

**P* < 0.05; DDS: dietary diversity score; CI: confidence interval; RR: risk ratio; CDD: changes in dietary diversity.

Model 1: no adjustment; Model 2: adjusted for age and sex; Model 3: adjusted for age, sex, residential location, education status, body mass index, drinking status, smoking status, exercise status, marital status, financial support, and history of chronic disease.
